# Examining the prevalence and predictors of anxiety and depression across treatment stages in prostate cancer: a systematic review

**DOI:** 10.3332/ecancer.2025.2041

**Published:** 2025-11-19

**Authors:** Oluwafemi E Adesina, Oluwadamilare Akingbade, Emmanuel O Adesuyi, Yetunde Tola, Ooreofe Bolanle Adeyemi, Tosin Akintunde, Stephan Osei, Julius Maitanmi, Deborah T Esan

**Affiliations:** 1 Institute of Nursing Research Nigeria, Osogbo 232111, Nigeria; 2 University of Alberta, Edmonton, AB T6G 2R3, Canada; 3 Birmingham City University, B4 7RJ Birmingham, UK; 4 School of Nursing, Dalhousie University, B3H 4R2, Canada; 5 Hohai University, Nanjing, 211100 China; 6 Babcock University, Ilishan 121103, Nigeria; 7 Bowen University, Iwo, Osun 220103, Nigeria; a https://orcid.org/0009-0005-7567-3431; b https://orcid.org/0000-0003-1049-668X; c https://orcid.org/0000-0003-0214-6401; d https://orcid.org/0000-0001-9408-0694; e https://orcid.org/0000-0001-6039-9568; f https://orcid.org/0000-0001-9136-6603; g https://orcid.org/0000-0002-3896-8207

**Keywords:** anxiety, depression, prostate cancer, prostatic neoplasm, prevalence, predictors

## Abstract

Anxiety and depression are common in prostate cancer (PCa) patients and negatively impact the quality of life, treatment outcome, survival and overall well-being, thus, requiring interventions to meet the psychosocial needs of PCa patients across treatment stages. However, there is not enough information to guide the design of these interventions, as there are still areas of lack of clarity regarding the prevalence and predictors of anxiety and depression in PCa patients. Therefore, this review was conducted to examine the literature to identify the overall prevalence of anxiety and depression across treatment stages in PCa patients and to identify the predictors of anxiety and depression in this population. A literature search was conducted from the Cochrane library, Ovid Medline and APA PsycINFO databases. Eighteen eligible studies were included in the final review. The findings were analysed using a narrative synthesis. The study quality was assessed using the Joanna Briggs Institute critical appraisal checklist. The prevalence of anxiety and depression was found to be between 6% to 44.8% and 10% to 48%, respectively. Notably, the prevalence of depression was higher in the post-treatment phase than in the treatment phase. Finally, the result demonstrates that socio-economic/demographic, clinical and lifestyle factors determine patients’ predisposition to anxiety and depression. These demonstrate that the prevalence of anxiety and depression is high across the PCa disease trajectory and that some patients are more likely to experience anxiety and depression than others. Therefore, we recommend periodic assessment to identify at risk patients and those with clinically significant or worsening levels of anxiety and depression for timely interventions to mitigate the risks or ameliorate the symptoms of anxiety and depression.

## Introduction

Globally, prostate cancer (PCa) is the leading type of cancer in men Global Cancer Observatory [19]. As of 2020, it accounted for 7.3% of all cancers diagnosed worldwide and is implicated in 3.8% of every cancer-related death recorded [19]. The incidence and prevalence of PCa have witnessed a steady rise over the years, increasing by 3% annually between 2014 and 2019 in the US [45].

By 2020, 1 in every 1,000 cancers diagnosed in the USA was PCa, thus surpassing lung cancer to become the second most diagnosed cancer in the USA [49]. In the same year, PCa surpassed breast cancer in the UK to become the most diagnosed cancer type, with PCa accounting for 12.4% of all new cases [9, 20].

The PCa disease trajectory consists of three time-points: pre-treatment, on-treatment and post-treatment/survival phase. The whole diagnosis and treatment journey is an emotional experience for several reasons, including the shock of the diagnosis, thoughts of the possibility of death, uncertainty about treatment outcome, impaired sexuality and a decline in quality of life [43], which affects the psychological wellbeing of patients.

Evidence shows that anxiety and depression are common in PCa, with prevalence rates higher than in the general population [27] and vary according to the phase of treatment the patient is in (2019). Both disorders have been strongly linked with poor adherence to treatment, poor treatment outcomes and increased risk of suicide among PCa patients [8, 46], which highlights a crucial clinical problem requiring the development of interventions to address.

To plan an effective intervention, understanding the factors that predict anxiety and depression among PCa patients is essential. In addition, a clear understanding of where a patient is more likely to experience anxiety and depression in the PCa disease trajectory is also crucial. However, these remain unclear.

To date, one published systematic review by Watts *et al* [52] examined the prevalence of anxiety and depression across the PCa disease trajectory. The review found the prevalence of anxiety and depression to range from 15% to 27%, with higher rates in the post-treatment phase than during treatment.

However, since the last systematic review was published (2014) nearly a decade ago, several primary studies have emerged on this topic, all reporting varying prevalence rates across the PCa disease trajectory. For instance, Erim *et al* [14, 15] reported that the prevalence of depression was lower in the post-treatment stage compared to the treatment stage, yet others argue it is higher in the post-treatment stage [13]. Additionally, previous studies, including the systematic review by Watts *et al* [52], did not explore the predictors of anxiety and depression across the PCa disease trajectory. Thus, an updated systematic review is warranted.

The current study aims to systematically analyse the literature to ascertain the current prevalence rates of anxiety and depression in PCa patients across the treatment and post-treatment phases of PCa. Second, the review aims to examine the potential predictors of anxiety and depression in this patient group.

## Methods

### Protocols and registration

The systematic review was conducted using the Preferred Reporting Items for Systematic Reviews and Meta-analyses (PRISMA) guidelines. The review protocol was registered with PROSPERO (registration number: CRD42023469982).

### Eligibility criteria

The CocoPop framework [34] was used to frame the research question and eligibility criteria. It consists of three elements: 1) the disease condition being studied, 2) the context of its occurrence and 3) the characteristics of the population it is being studied in [34]. The framework applied to this current review can be summarised as follows:

- Condition: Studies that examined the prevalence and predictors of anxiety and depression in PCa patients were included in the review.
- Context: Studies examining patients in the on-treatment and post-treatment/survival phases of the PCa disease trajectory were also included.
- Population: Studies involving men aged 15 and above diagnosed with PCa were included, as evidence shows an increasing prevalence of PCa in adolescents and young adult males [5].

### Search strategy

A systematic search was conducted on the Cochrane Library, APA PsycINFO and Ovid Medline to identify studies that reported the prevalence of anxiety and/or depression in individuals diagnosed with PCa across the treatment stages of the disease. The details of the search strategy are presented in Table 1. The databases were searched from 2013 to June 2023. Also, to supplement the search, the reference lists of included studies were screened to identify studies not initially retrieved from the database search.

### Article screening and selection process

**Figure 1.**
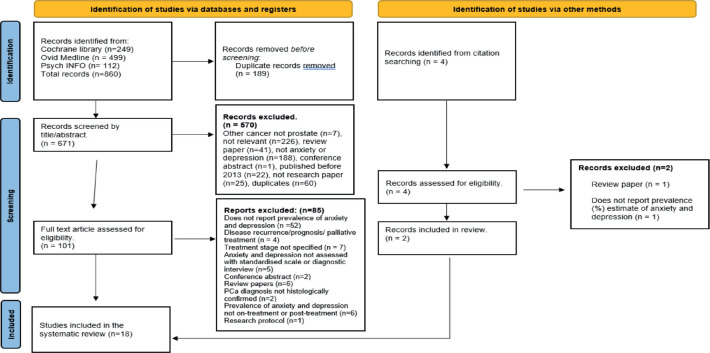
Data base search and article selection.

Studies retrieved from the database search were de-duplicated using Endnote 20, after which Oluwafemi E Adesina (OEA) and Akingbade Oluwadamilare (AO) independently screened the articles. First, the title and abstract of the articles were screened against the inclusion and exclusion criteria. Next, the full texts of potentially eligible studies were retrieved and further screened. Throughout the review process, conflicts were discussed and resolved through consensus. The article screening and selection process is summarised using a PRISMA flow diagram in Figure 1.

### Risk of bias assessment

OEA and AO assessed the methodological quality of the included studies using the Joanna Briggs Institute (JBI) critical appraisal checklist [34] as summarised in Table 2. Also, a scoring system was used to rate the studies as having low, moderate or high quality based on the overall score. Similarly, where there were disagreements, they were resolved by discussing with another team member. The scoring and quality rating criteria are summarise in Supplementary Tables 1 and 2.

### Data extraction and analysis

Further, the two reviewers independently conducted the data extraction from the included studies, using a modified JBI data extraction form. The following data were extracted: sample size, age, treatment received, treatment stage, the instrument used to evaluate anxiety and depression, study design, prevalence of anxiety and/or depression and the predictors of anxiety and/or depression.

Finally, a narrative synthesis was conducted to analyse the key findings of the review, particularly the prevalence and predictors of anxiety and depression across the treatment stages. While statistical pooling of prevalence estimates and meta-analysis were considered, the wide variation in the study designs, instrument for data collection and cut-off scores for determining caseness precluded these. Therefore, a narrative review/synthesis was adopted.

Missing or incomplete data were retrieved by contacting the study authors. Where this was not possible, the available findings were described and the assumptions made about the missing data were clearly stated.

## Results

After the database search and article screening process, 18 eligible studies were included in the review. The article screening and selection process is summarised in Figure 1. The studies included in the review examined the prevalence of anxiety and/or depression in PCa patients in the on-treatment and/or post-treatment phase. The characteristics of the studies included in this review are summarised in Table 3.

The included studies were conducted in a range of countries, including China (*n* = 2), Australia (*n* = 2), the Netherlands (*n* = 1), UK (*n* = 1), Republic of Ireland and UK (*n* = 2), USA (*n* = 4), Italy (*n* = 2), Taiwan (*n* = 1), Japan (*n* = 1) and Spain (*n* = 2). All the studies were published between 2013 and 2021, with a pooled sample size of 21,718.

Further, participants’ mean age was reported in 13 studies [7, 10, 11, 14, 21, 23, 37, 41, 47, 48, 50, 51, 55] and it varied from 62.5 to 76. The mean age of all the participants across the 13 studies was 68.73 years.

Notably, two treatment stages were identified in this review: the on-treatment stage and the post-treatment/ survival stage. Most of the studies (*n*= 15) assessed anxiety or depression or both in the post-treatment phase of PCa only, one assessed depression in the on-treatment phase only [55], while two assessed the same outcome in both the on-treatment and the post-treatment phase [39, 41] and one study assessed depression in the on-treatment stage alone [55]. Seven studies reported the prevalence rates of both anxiety and depression [11, 23, 37, 40, 42, 48, 51], eight reported rates of only depression [1, 4, 7, 10, 21, 39, 41, 55] and three reported rates of only anxiety [14, 47, 50]. Regarding the predictors of anxiety and depression, only nine studies reported them [4, 10, 11, 39, 40, 42, 48, 51, 55].

The cancer treatments received by the participants were reported in all but one study [42]. The treatment modalities reported across the six studies are radical prostatectomy/surgery (*n* = 12) [1, 7, 21, 23, 37, 39, 40, 47, 48, 50, 51, 55], radiotherapy (*n* = 9) [1, 4, 11, 21, 39, 40, 47, 50, 51], ADT/hormone therapy (*n* = 8) [4, 10, 11, 39, 41, 51, 55] and chemotherapy (*n* = 1) [4]. The most common treatment that patients received was surgery/radical prostatectomy [1, 48, 50, 51, 55], while the least identified treatment received by patients was chemotherapy [4].

Most studies (*n* = 10) reported the prevalence of anxiety, all of which were assessed in the post-treatment phase [11, 14, 23, 37, 40, 42, 47, 48, 50, 51]. The rates varied across these studies, ranging from 6% to 44.8%. Also, no study in the review assessed anxiety prevalence in the on-treatment stage.

For depression, 12 studies reported the prevalence in the post-treatment phase of PCa alone [1, 4, 7, 10, 11, 21, 23, 37, 40, 42, 48, 51], two reported depression prevalence in both the on-treatment and post-treatment phase [39, 41], while one reported depression prevalence in the on-treatment phase only, at multiple times and across three sub-groups [55] as shown in Table 3.

The prevalence of depression varied across all the studies, ranging from 9.1% to 46.9% in the on-treatment phase and 7% to 48% in the post-treatment phase.

The predictors of anxiety and depression were reported in only nine of the studies, with seven of these studies assessing PCa patients in the post-treatment phase [4, 10, 11, 41, 42, 48, 51], one in the on-treatment phase [55] and the last across both the on-treatment and post-treatment phases [39]. The predictors of anxiety and depression identified in the current review can be categorised into three domains: socio-economic factors, clinical factors and lifestyle factors. These predictors include age, marital status, level of education, employment status, living in Northern Ireland, a history of depression, cancer symptoms at diagnosis, comorbidities, treatment modality, post-operative erectile dysfunction, smoking, alcohol consumption and inadequate physical activity.

## Discussion

This review was conducted to identify the overall prevalence as well as the predictors of anxiety and depression in PCa patients across treatment stages. The findings offer crucial insight into the psychosocial needs of PCa patients and how to best support them, as discussed subsequently.

### Prevalence of anxiety and depression

The prevalence of anxiety varied across the ten studies that reported it. Notably, the reported prevalence of anxiety covered only the post-treatment stage, as none of the studies assessed or reported the prevalence of anxiety in the on-treatment stage. The post-treatment prevalence ranged from 6% to 44.8%. This finding is not unexpected, as a similar range of anxiety prevalence was reported in previous studies [27, 54], confirming that PCa patients still experience high anxiety levels after completing treatment. It also suggests that the psychosocial needs of PCa patients in these stages of treatment are not being met.

The variation observed in the prevalence of anxiety may be due to the use of different instruments with different cut-off scores to assess anxiety and varying sample size [52], as the studies in this review reporting the highest prevalence of anxiety had low to moderate sample sizes [23, 47].

Finally, the study location may also explain the difference observed in the prevalence rate of anxiety. The two studies included in this review with the highest anxiety prevalence (41.7% and 44.8%) were conducted in Asia [23, 47]. Another study conducted in China, an Asian country, found the prevalence of anxiety in PCa patients to be 43.2% [54]. This observation strengthens the argument that there is a possible link between the study location and the variation in anxiety prevalence reported. It also suggests that Asian men with PCa might be more susceptible to anxiety than their European counterparts. However, more empirical studies are required to validate this view.

This review found the prevalence of depression to be between 7% and 48%, a rate unexpectedly higher than what was previously reported in the literature (8.2% to 25%) [3, 16, 27]. This may be due to the use of different instruments to assess depression, as it has been demonstrated that the use of different instruments in assessing depression accounts for the difference in the reported prevalence [44].

Furthermore, the difference in treatment type received in different sample populations could also explain the variation observed in the reported depression prevalence. The current review found that in most of the studies that reported the highest levels of depression, the participants were primarily treated with ADT. In contrast, the other studies that reported low to moderate rates of depression had patients who received other treatments (i.e., prostatectomy and RT), with or without ADT. This is corroborated by a previous finding that ADT is linked to a higher risk of depression [18].

The prevalence of depression, but not anxiety, is higher after treatment than during treatment. This result is corroborated by Watts *et al* [52]’s meta-analysis result, which showed that the overall prevalence of anxiety and depression among PCa patients in the post-treatment stage was higher compared to the treatment stage. A plausible explanation for this pattern is that treatments for PCa result in debilitating after-effects such as impaired sexual function, impaired bowel and urinary function, which can all negatively impact patients’ mental health [27]. Additionally, fear of cancer recurrence is a common concern among PCa patients [28] and may also contribute to the increased level of anxiety and depression experienced by PCa patients in the post-treatment stage. Thus, highlighting the need for targeted psychosocial interventions in this treatment phase of PCa.

Conversely, Erim *et al* [14, 15] found the prevalence of depression to decline in the post-treatment phase. However, they did not compare on-treatment rates to the post-treatment rates. Instead, the authors compared pre-treatment rates and post-treatment rates. Based on their findings, no conclusion can be made regarding the level of depression experienced by PCa patients during treatment compared to after treatment. Therefore, this review confirms that the prevalence of depression in PCa patients is higher in the post-treatment phase compared to the treatment phase.

Notably, none of the studies in this review reported the prevalence of anxiety in the treatment phase. Thus, making it difficult to identify the difference in the anxiety levels experienced by patients while on treatment compared to after treatment. However, the review of Watts *et al* [52] shows a similar trend of fewer studies in the on-treatment stage. For instance, of the 27 studies included in the review, only four assessed anxiety in the on-treatment stage and of the four, two were studies that assessed patients on active surveillance.

### Predictors of anxiety and depression

#### Socio-economic factors

Regarding the predictors, being younger was found to predict both anxiety and depression in PCa patients. This corroborates the result of two studies that assessed anxiety in PCa and ovarian cancer patients [24, 29] and debunks the finding of Yu and Li [54], which suggests that older age predicts anxiety. Younger PCa patients are more likely than their older counterparts to experience anxiety and depression because older age is associated with higher levels of resilience [38], which is believed to ameliorate the impact of stressful life events on psychological well-being [30]. Thus, it can be inferred that younger PCa patients would benefit from interventions aimed at improving resilience.

Furthermore, being unmarried predicted both anxiety and depression in PCa patients. This confirms previous findings that reveal that divorced and unmarried PCa patients are more likely to experience anxiety and depression than their married counterparts [36, 53]. It is possible that being married provides the needed psychosocial support to cope with the distress of PCa. Therefore, this highlights a need for alternative psychosocial support systems, such as support groups for unmarried PCa patients.

Additionally, educational attainment predicts the occurrence of anxiety and depression. Less educated PCa patients were more likely to experience anxiety and depression than the more educated ones. This finding is echoed by other studies that have previously demonstrated the link between educational attainment and anxiety [40, 54]. Besides, a low level of education is associated with unemployment and consequent financial difficulty [33]. Similarly, the physical impact of PCa symptoms and treatment after-effects, such as fatigue, can affect patients’ ability to work [6], resulting in low or no income. The ensuing financial difficulty predisposes patients to depression [25, 26].

#### Clinical factors

It was observed that medical comorbidities increased the likelihood of developing anxiety and depression in PCa patients [11, 42, 51]. This is possibly owing to the correlation between chronic illnesses and anxiety [28], as the comorbidities identified in this review were primarily chronic illnesses such as hypertension, chronic obstructive pulmonary disease, asthma, diabetes, arthritis, inflammatory bowel disease and so on [51]. Given that PCa can also independently contribute to the experience of anxiety and depression among patients [27], it is difficult to establish if the anxiety experienced is attributable to PCa itself or the comorbidities. This highlights a need for longitudinal controlled studies to compare the level of anxiety in PCa patients with comorbidities and those without co-morbidities to ascertain if co-morbidities indeed predict anxiety in PCa.

Furthermore, treatment modalities, particularly receiving ADT and open robotic surgery, have been shown to predict depression and not anxiety. This is because of the after-effects of treatments. For instance, ADT causes breast tenderness, impaired sexual function, weight gain, hot flushes and irritability [35, 37]. Similarly, prostate surgery results in erectile dysfunction and impaired urinary function [54] and all these put the PCa patient at a higher risk of depression. Since these are inevitable side effects of treatments, their impact on a patient’s mental health can only be minimised. This can be achieved by equipping patients with adequate information about treatment and side effects before treatment and providing ongoing support while on treatment and even after treatment.

In conclusion, since these clinical predictors are easy for clinicians to spot, they offer a promising avenue to identify and provide prompt management for PCa patients at risk of experiencing anxiety/depression.

#### Lifestyle factors

This review found smoking and alcohol consumption to predict depression but not anxiety in PCa patients [55]. It is thought that depressed PCa patients may resort to smoking as a coping mechanism. However, the causal links between them remain unclear [17]. Similarly, it is thought that PCa patients consume alcohol as a coping mechanism for the distress of the illness. A previous study suggested a causal relationship between alcohol use and depression, stating that alcohol use increases the risk of depression [32]. Thus, highlighting the need to pay attention to the alcohol use pattern in PCa patients, as it can provide an avenue for early detection and prevention of depression.

Also, reduced physical activities predicted depression but not anxiety among PCa patients [11]. Several reviews also found that engaging in exercise can prevent or improve the symptoms of depression in cancer patients [22, 31] and is most effective in the on-treatment phase. Given these, it could be inferred that incorporating exercise in managing PCa patients would prevent or reduce the risk of depression.

Finally, seven of the studies in which these predictors were identified assessed PCa patients in the post-treatment phase, while one assessed the patients in the on-treatment phase [55], and the last assessed them across both the on-treatment and post-treatment phases [39]. This uneven distribution of studies across treatment stages makes it difficult to ascertain which predictors may account for the different levels of anxiety and depression experienced in the treatment and post-treatment phases of PCa.

#### Strengths and limitations of the study

On the one hand, this systematic review has some strengths. First, all the included studies utilised a standardised instrument to assess anxiety and depression. Also, the methodological quality of the included studies was assessed utilising an appraisal tool that has been previously tested.

On the other hand, owing to the wide variation in the study designs, instruments for data collection and cut-off scores for determining caseness, a meta-analysis or a pooled prevalence estimate was not feasible. Instead, a narrative synthesis was conducted.

Further, there was a paucity of on-treatment studies included in the review because most studies utilised a cross-sectional design, which measured point prevalence and, thus, was not a suitable design to measure changes in the prevalence of anxiety and depression over time. Furthermore, this current review did not count studies on active surveillance as on-treatment studies. This is because active surveillance is considered a management approach that monitors rather than actively treats a disease [2, 12]. Hence, active surveillance studies were excluded from this review.

Notwithstanding, these limitations do not undermine the findings of this review. They only highlight areas readers need to consider while interpreting the results.

#### Recommendations for future research

There is a paucity of research that assessed anxiety and depression in PCa patients in low and middle-income countries (LMICs), especially in Africa and the Caribbean, geographical regions that have the largest population of black men who are the most affected by PCa. Thus, highlighting a crucial research gap and a need to conduct quality studies in LMICs.

Finally, future research assessing anxiety and depression across treatment stages should utilise longitudinal designs to address the limitations of cross-sectional designs.

#### Implications for clinical practice

Given the findings, it is recommended that an assessment of anxiety and depression should be conducted pre-treatment (as a baseline for subsequent evaluations), during treatment and after treatment to identify those with clinically significant or worsening levels of anxiety and depression for timely referral for appropriate mental healthcare. Preferably, the assessment tool should be concise, easy to use and have an empirically proven internal validity, such as the HADS, MAX-PC or PHQ-9 [40].

Further, cancer multi-disciplinary teams could utilise the supportive evidence provided in this study in designing guidelines and pathways for the screening, stratification and management or referral of at-risk patients.

Considering that anxiety and depression persist into the PCa survivorship phase, there is a clear need to provide ongoing patient support post-treatment through support groups and uro-oncology clinical nurse specialist (CNS) clinics. Although in developed countries, CNS clinics and PCa support groups are available and have proven useful, it would be beneficial to incorporate more programs aimed at improving resilience, as high levels of resilience can serve as a buffer against anxiety and depression [38].

Finally, as evidence suggests that the lack of adequate physical activity increases the risk of experiencing depression in PCa patients, it may be worthwhile to incorporate supervised exercise in the management of PCa, as it has been demonstrated to prevent and improve depression symptoms in cancer patients [22].

## Conclusion

This review confirms that anxiety and depression prevalence are high across the PCa disease trajectory, and depression prevalence is higher in the post-treatment phase. It also demonstrates that socio-economic/demographic, clinical and lifestyle factors determine patients’ predisposition to anxiety and depression. This suggests that some PCa patients are more likely to experience anxiety and depression than others. Therefore, we recommend periodic screening to identify patients with clinically significant or worsening levels of anxiety and depression for timely interventions. Finally, incorporating exercise and supportive care via CNS clinics and PCa support groups are likely to reduce the risk of or ameliorate anxiety and depression symptoms.

## List of abbreviations

PCa, Prostate cancer; CRUK, Cancer Research UK; PRISMA, Preferred Reporting Items for Systematic Reviews and Meta-analaysis; PROSPERO, International Prospective Register of Systematic Reviews.

## Conflicts of interest

The authors have no conflicts of interest to declare.

## Funding

No funding was received for this work.

## Figures and Tables

**Table 1.** Database search terms.

OR		AND		AND	OR	
Anxiety	Depression		PCa		Treatment	Survival
“anxiety” OR “anxi*”	“depress*” OR “low mood” OR “Depression” OR “cancer-related distress” OR “Psychological issues”	“PCa” OR “prostatic cancer” OR “prostate carcinoma” OR “cancer of the prostate” OR “prostate neoplasm” OR “Prostatic neoplasm” OR “Prostate tumour” OR “Prostate Malignancy”	“PCa chemotherapy” OR “PCa Chemo*” OR “PCa radiotherapy” OR “PCa brachytherapy” OR “PCa immunotherapy” OR “prostate chemoradiotherapy” OR “hormone deprivation therapy” OR “Androgen deprivation therapy” OR “anti-androgen therapy” OR “Prostatectomy” OR “prostate resection” OR “Prostate Surgery” OR “castration”	“PCa survivor” OR “Living with PCa” OR “PCa follow-up”

**Table 2.** Summary of the methodological quality appraisal using the JBI critical appraisal checklist.

S/N	JBI critical appraisal checklist question	Tavlarides et al [48]	Sharp et al [42]	Bensley et al [4]	Zhang et al [55]	Venderbos et al [50]	Baden et al [1]	Watson et al [51]	Saini et al [39]	Hoyt and Carpenter [21]	Punnen et al [37]	Chen et al [10]	Hu et al [23]	Taoka et al [47]	Sánchez Sánchez et al [40]	Erim et al [15]	Sánchez Sánchez et al [40]	Chipperfield et al [11]	Boeri et al [7]
**1**	Was the sample frame appropriate to address the target population?	Y	Y	Y	Y	Y	Y	Y	Y	Y	Y	Y	Y	Y	Y	Y	Y	Y	Y
**2**	Were study participants sampled in an appropriate way?	Y	Y	Y	Y	Y	Y	Y	Y	Y	Y	Y	Y	Y	Y	Y	Y	Y	Y
**3**	Was the sample size adequate?	Y	Y	Y	Y	Y	Y	Y	Y	Y	Y	Y	U	U	Y	Y	U	Y	Y
**4**	Were the study subjects and the setting described in detail?	Y	Y	Y	Y	Y	Y	Y	Y	Y	Y	Y	Y	Y	Y	Y	Y	Y	Y
**5**	Was the data analysis conducted with sufficient coverage of the identified sample?	Y	Y	Y	Y	Y	Y	Y	Y	Y	Y	Y	Y	Y	Y	Y	Y	Y	Y
**6**	Were valid methods used for the identification of the condition?	Y	Y	Y	Y	Y	Y	Y	Y	Y	Y	Y	Y	Y	Y	Y	Y	Y	Y
**7**	Was the condition measured in a standard, reliable way for all participants?	Y	Y	N	Y	Y	Y	Y	Y	Y	Y	Y	Y	Y	Y	Y	Y	Y	Y
**8**	Was there appropriate statistical analysis?	Y	Y	Y	Y	Y	Y	Y	Y	Y	Y	Y	Y	Y	Y	Y	Y	Y	Y
**9**	Was the response rate adequate, and if not, was the low response rate managed appropriately?	N	Y	Y	Y	Y	Y	Y	Y	U	Y	N	U	U	Y	Y	Y	Y	Y
	Overall score	8	9	8	9	9	9	9	9	8	9	8	7	7	9	9	8	9	9
	Quality rating	Good	Good	Good	Good	Good	Good	Good	Good	Good	Good	Good	Good	Good	Good	Good	Good	Good	Good

**Table 3.** Summarising the characteristics of studies included in the review.

s/n	Study details (Author and location)	Sample size (n)	Study design	Age (mean/SD/frequency)	PCa treatment received	Treatment stage	Anxiety and depression outcome measure	Prevalence of reported anxiety	Prevalence of reported depression	Identified predictors of anxiety and depression
**1**	Tavlarides *et al* [48] (USA)	365	Cross-sectional design	63.9	Radical prostatectomy	Post-treatment	MAX-PC (cut off 27) and EPIC	6%	48%	Not reported.
**2**	Sharp *et al* [42] (RoI and UK)	3,348	Cross-sectional design	24.8%≤ 59 years. 49.1%- 60–69 years 26.1% > 70	Not specified	Post-treatment	DASS Cut off 10 for depression subscale. Eight for the anxiety subscale	16%	17%	Age, education, marital status, employment status, co-morbidity, previous history of depression, living in NI
**3**	Bensley *et al* [4] (Australia)	9,712	Retrospective cross-sectional design	40% <65 years. 60% ≥ 65 years.	Radiation therapy, ADT, chemotherapy,	Post-treatment.	EPIC-26	Not assessed	10%	Treatment modality (receiving ADT) and Age.
**4**	Zhang *et al* [55] (China)	146	Prospective longitudinal controlled study.	ADT- 71.6, RP-69.37, BPH- 70.16	ADT and radical prostatectomy	On treatment.	Zung’s SDS scale. Cut off 40	Not assessed	Month 9: 16.3% Month12: 28.3% Month15: 26.5%	Alcohol consumption and smoking
**5**	Venderbos *et al* [50] (Netherlands)	879	Cross-sectional design.	RP- 76, RT- 74.5, AS – 71.9, Ref. group- 74.5	Radical prostatectomy and radiation therapy.	Post-treatment	STAI-6 Cut off score 44	13% in RP and 12.3% in RT	Not assessed	Not reported.
**6**	Baden *et al* [1] (ROI and UK)	3,348	Cross-sectional design.	23.9%<60, 48.7% (60–59), and 27.4%>70 years.	Radical prostatectomy and radiation therapy.	Post-treatment	DASS 21 Cut off score 10	Not assessed	14.4%	Not reported
**7**	Watson *et al* [51] (UK)	316	Cross-sectional design.	67.8 years	Surgery, radiotherapy and hormone therapy.	Post-treatment	HADS Cut off 11	17%	10%	Co-morbidities
**8**	Saini *et al* [39] (Italy)	103	Cross-sectional design	Median age = 73	RT, RP, and ADT	On-treatment (ADT group) and post-treatment (RT and RP group in follow-up)	HADS Cut-off score of 7	Not reported	On treatment- 46.9% Post-treatment – 18.5%	ADT
**9**	Hoyt and Carpenter [21] (USA)	66	Cross-sectional design	65.76	RP and RT	Post-treatment	CESD Cut-off score of 16	Not reported	23%	No data.
**10**	Punnen *et al* [37] (USA)	679	Prospective longitudinal cohort study	60	RP	Post-treatment	PHQ-9 (cut off 5) GAD-7 (cut off 5)	15%	15%	No data.
11	Chen et al [10] (Taiwan)	71	Retrospective cross-sectional design	72.5	ADT	Post-treatment	PHQ-9 Cut	No data	41%	Longer duration of treatment with ADT.
**12**	Hu *et al* [23] (China)	194	Prospective longitudinal cohort study	62.5	RP	Post-treatment	Zung’s SAS/SDS Cut off for both subscales 50	44.8%	34.0%	No data
**13**	Taoka *et al* [47] (Japan)	70	Cross-sectional design	68.7	RP and RT	Post-treatment	STAI. Cut-off not reported	41.7%	No data	No data
**14**	Sánchez Sánchez *et al* [40]	184	Cross-sectional design	Median age 71	RP, RT, and hormone therapy	Post-treatment	HADS scale. Cut off score 11	14.1%	7%	Level of education
**15**	Erim *et al* [15] (USA)	1,016	Cross-sectional design	65		Post-treatment	MAX-PC Cut off 27	8.0	No data	No data
**16**	Sánchez Sánchez et al [40] (Spain)	33	Prospective longitudinal design	70.8	ADT	On-treatment and post-treatment	GDS. Cut off score 5.	No data	9.1% on-treatment and 30.3% post-treatment	No data
**17**	Chipperfield *et al* [11] (Australia)	377	Cross-sectional design	67.6	ADT and radiotherapy	Post-treatment.	HADS cut-off score 8	13.6%	19.6%	Comorbidity, younger age and inadequate physical activity per NPAGA.
**18**	Boeri *et al* [7] (Italy)	811	Prospective longitudinal design	64.7	Prostatectomy	Post-treatment	BDI cut off 11	No data	26.3% at 6 months 36.7% at 36 months.	Age, open robotic prostatectomy, and post-operative erectile dysfunction.

ADT: androgen deprivation therapy

## Supplementary tables

**Supplementary Table 1.** The scoring criteria used to determine the methodological quality of included studies.

s/n	Checklist question response	Score assignment
1	Yes (Y)	1
2	No (N)	0
3	Unsure (U)	0
4	Not applicable (N/A)	0

**Supplementary Table 2.** Quality rating of included studies per overall score.

S/N	Overall score	Quality rating
1	0–3	Low quality
2	4–6	Moderate quality
3	7–9	High quality
